# Gut microbiota in women with gestational diabetes mellitus has potential impact on metabolism in pregnant mice and their offspring

**DOI:** 10.3389/fmicb.2022.870422

**Published:** 2022-08-05

**Authors:** Shengtang Qin, Yutong Wang, Shuxian Wang, Bohan Ning, Jing Huai, Huixia Yang

**Affiliations:** ^1^Department of Obstetrics and Gynecology, Peking University First Hospital, Beijing, China; ^2^Beijing Key Laboratory of Maternal Fetal Medicine of Gestational Diabetes Mellitus, Beijing, China; ^3^Department of Pathology, Peking University First Hospital, Beijing, China

**Keywords:** fecal microbiota transplantation, gestational diabetes mellitus, germ-free mice, offspring, gut microbiota, pregnancy

## Abstract

Studies have shown that gestational diabetes mellitus (GDM) is closely related to abnormalities in the gut microbiota, and the offspring of these women have an increased risk of diabetes. There is no direct evidence of whether bacteria in women with GDM colonize the intestinal tract of offspring and cause hyperglycemia. In this fecal microbiota transplantation (FMT), pregnant mouse model study, two groups of germ-free (GF) mice after FMT showed different colonization patterns of gut microbiota and phenotype. Compared with the control group (healthy-FMT), we found in the GDM-FMT group as a lower relative abundance of *Akkermansia* and *Faecalibacterium*; a lower content of short-chain fatty acids and naringenin in feces; an elevated blood glucose; an inflammatory factor expression (TNF-α, CXCL-15, and IL-6), and a hepatic fat deposition. In addition, the influence of the gut microbiota continued in offspring. The gut microbiota of the offspring of GDM-FMT mice was still different from that of the control group as a lower relative abundance of *Akkermansia* and *Parvibacter*; and a higher relative abundance of bacteria such as *Oscillibacter*, *Romboutsia*, and *Harryflintia*. In addition, the offspring of GDM-FMT mice had higher body weight and blood glucose levels than the control offspring.

## Introduction

Gestational diabetes mellitus (GDM) is defined as carbohydrate intolerance of variable severity with onset that occurs during pregnancy. In the recent years, the incidence of GDM has been increasing year by year. According to the reports, the prevalence of GDM is between 9.3 and 25.5% in the global population (Sacks et al., 2012). A survey of 13 hospitals in China in 2013 showed that the incidence of GDM had reached 17.5% (Zhu et al., 2013). Gestational diabetes mellitus has become an important pregnancy disease, as it is associated with poor fetal–maternal outcomes, including polyhydramnios, preeclampsia, shoulder dystocia, and macrosomia (Beucher et al., 2010; Ismail et al., 2011; Catalano et al., 2012; Kc et al., 2015). The vast majority of diabetic patients will return to normal blood glucose levels after delivery. Even so, the risk of developing type 2 diabetes within 10–15 years after delivery was still 40% higher than that of non-gestational diabetes mellitus (non-GDM) women (Lauenborg et al., 2004; Damm et al., 2016).

The interaction between the gut microbiota and the natural intestinal immune system is considered to be an exogenous factor that can affect susceptibility to diabetes. The relationship between gut microbiota and diabetes has been widely studied because intestinal bacteria express microbe-associated molecular patterns (MAMPs) that can activate Toll-like receptors (TLRs). In the pathogenesis of GDM, the TLRs can cause protein inflammation and activate the nuclear factor-κB (NF-κB) pathway (Xie et al., 2014). A high-fat diet could increase the abundance of lipopolysaccharide-producing (LPS-producing) bacteria in the gut to increase fat deposition, increase body fat, induce excessive fasting blood sugar, and cause inflammation (Cani et al., 2008; Caesar et al., 2015). Changes in the gut microbiota during pregnancy lead to increased production of short-chain fatty acids (SCFAs), which can inhibit the production of the proinflammatory cytokines interleukin-6 (IL-6) and tumor necrosis factor-α (TNF-α) (Roelofsen et al., 2010). In addition, SCFAs can promote insulin secretion, increase the number of β-cells (Fuller et al., 2015), activate catabolic pathways (Tilg and Moschen, 2014; Paul et al., 2018), and affect the formation of the immune system of offspring (Martin et al., 2010; Romero and Korzeniewski, 2013; Gomez de Aguero et al., 2016). Therefore, changes in the composition of the intestinal microbiota will affect host metabolism and abnormal blood glucose regulation (Geurts et al., 2014).

Several studies discovered that the gut microbiota composition of women with GDM was different from that of women without GDM (Sililas et al., 2021), and this may be closely related to the pathogenesis of GDM (Koren et al., 2012; Mokkala et al., 2017; Crusell et al., 2018). Different studies have different interpretations of the abundance of gut microbiota in women with GDM and have explored many related bacteria, including bacteria that were positively [*Bacteroides dorei* (Wu et al., 2020), *Blautia* (Liu et al., 2020), *Klebsiella variicola* (Kuang et al., 2017), Ruminococcaceae (Mokkala et al., 2017)] and inversely [*Bifidobacterium* (Kuang et al., 2017), *Akkermansia* (Everard et al., 2013; Liu et al., 2020; Shih et al., 2020), *Faecalibacterium* (Tilg and Moschen, 2014)] related to GDM. These bacteria are closely related to the production of short-chain fatty acids, intestinal and peripheral tissue inflammation, glucose and lipid metabolism, and insulin resistance (Macfarlane and Macfarlane, 2003; Hansen et al., 2012).

In 2006, the United Nations Standing Committee on Nutrition proposed that the most critical period to determine the nutritional and health status of children is from pregnancy (9 months *in utero*) to 1–2 years after birth (1,000 days in early life), which is the most critical period to determine the nutritional and health status of a person’s life (Dora et al., 2015). Therefore, how to form a well-distributed intestinal microenvironment in the early stages of life is very important. Microorganisms in the gut have difficulty surviving outside the host. A person’s own gut microbiota colonization is mostly derived from direct transmission by others, and most come from their own mothers (Crusell et al., 2020; Selma-Royo et al., 2021). The vertical transmission and colonization of infant intestinal microbiota is a gradual process. Any interference in this process may bring adverse consequences and affect its normal colonization and development of the microbiota. In the recent years, disorders of the intestinal microbiota have been considered to be a key factor in the occurrence of chronic inflammatory diseases such as irritable bowel syndrome and inflammatory bowel disease (Rautava et al., 2012; Belkaid and Hand, 2014), respiratory diseases (Gosalbes et al., 2013), and metabolic diseases. Therefore, it is very important to form a “healthy” gut microbial community early in life.

The offspring of women with GDM have an increased risk of diabetes (Khan, 2007; Farahvar et al., 2019; Sparks et al., 2022). Does this high risk of blood sugar abnormality come from the transmission of the mother’s abnormal microbiota? Studies have shown that gestational age at birth (La Rosa et al., 2014; Cong et al., 2016), delivery methods (Jakobsson et al., 2014), different feeding styles (breast milk or formula) (Liu et al., 2019), external environment, drug use, etc. can all affect the composition of infant intestinal microbiota (Rodriguez et al., 2015; Milani et al., 2017). Therefore, the influence of multiple factors provides no direct evidence that abnormal bacteria in pregnant women with gestational diabetes can be stably transmitted to offspring. We collected fresh feces of pregnant women with GDM, used fecal microbiota transplantation (FMT) to carry out intestinal microbiota transplantation experiments on GF mice to construct GDM model mice, explored the mechanism of abnormal glucose metabolism, and observed the bacteria of the offspring mouse group composition and growth and development, with an in-depth understanding of the impact of GDM-related bacteria on offspring.

## Materials and methods

### Animal procedures

In this study, animal tasks were approved by the Institutional Animal Care and Use Committee of Peking University First Hospital (J201884). Moreover, C57/BL6 GF mice (8 weeks old with a body weight 23.44 ± 3.52 g) were purchased from the Institute of Medical Laboratory Animals, Chinese Academy of Medical Sciences. All mice were housed in an isolated sterile environment. Mice were placed into individual cages with a 12-h:12-h dark:light cycle, a controlled temperature of 21 ± 4°C and 50–60% humidity, and *ad libitum* access to food and water. Mice were weighed every 3 days, and food intake was measured.

The fecal samples of the three individuals in the GDM donor group and the three individuals in the non-GDM donor group were mixed, diluted, and packed in an anaerobic incubator (100% N_2_). The mixture was suspended in 10 ml of PBS buffer and vortexed at room temperature for 5 min. After incubation for an additional 5 min, the supernatant was transferred to a new tub, mixed well, and loaded into anaerobic tube (500 μl per tube) and immediately stored at −80°C (Ridaura et al., 2013). The prepared anaerobic tube containing the suspension was placed in a transfer sleeve connected to the sterile isolator, sterilized by ethylene oxide (C_2_H_4_O) for 20 min, and then transferred to the sterile isolator.

Germ-free mice were orally inoculated with 200 μl of the fecal mixture from the GDM donor and healthy donor groups. All mother mice were reared in a germ-free environment until the end of the experiment. Some mice were sacrificed on the 21st day of gestation for the detection of inflammatory factors and immunohistochemistry. Two pregnant mice in each group were reserved for the offspring mice to continue the experiment. The offspring mice were separated from the mother mice 4 weeks after delivery and placed in an SPF environment. Fresh fecal samples from each mouse were collected with tweezers, placed in a sterile 1.5-ml tube, and immediately transferred to a −80°C freezer.

### Intraperitoneal glucose tolerance test and enzyme-linked immunosorbent assay test

Mice were fasted for 6 h and then given an intraperitoneal injection of glucose (2-g/kg body weight). The tail blood was collected at 0H, 0.5H, 1.5H, and 2H after injection, and blood glucose concentrations were measured with an Accu-Check glucometer (Roche, Germany, H87950). We sacrificed the mice on the 20th day of gestation to collect plasma. The concentrations of TNF-α, CXCL-15, and IL-6 in plasma were measured with an enzyme-linked immunosorbent assay hypersensitivity kit provided by JingLai Biotechnology Co., Ltd. according to the manufacturer’s instructions.

### 16S rRNA gene sequencing

The DNA was extracted with a QIAamp PowerFecal DNA kit (Qiagen, Hilden, Germany) following the manufacturer’s protocols, and the V3–V4 region of the 16S rRNA gene was amplified by polymerase chain reaction (PCR) with the forward primer 5′TCGTCGGCAGCGTCAGATGTGTATAAGAGACAGCCTA CGGGNGGCWGCAG3′ and reverse primer 5′GTCTCGTGGGCTCGGAGATGTGTATAAGAGACAGG ACTACHVGGGTATCTAATCC3′. The PCR products were sequenced on an Illumina HiSeq 2500 platform, and fast length adjustment of short reads (FLASH) was used to merge paired-end reads from the sequencing results (Magoc and Salzberg, 2011). Low-quality reads were filtered by the quality filter in the FASTX Toolkit 0.0.14. The analyzed data retained only high-quality (*Q* ≥ 25) reads with a base ratio greater than or equal to 90%, and chimeric reads were removed by USEARCH 64-bit v.8.0.1517. The number of reads for each sample was normalized based on the smallest sized sample by random subtraction. Operational taxonomic units (OTUs) were aligned by the UCLUST algorithm with 97% identity and taxonomically classified using the SILVA 16S rRNA database v.128. Alpha and beta diversities were generated in quantitative insights into microbial ecology.

### Untargeted metabolomics assays and quality control

Stool samples were quickly frozen in liquid nitrogen after collection and stored at −80°C until analysis. The metabolomics analysis was performed on a UHPLC system (1290 infinity LC, Agilent Technologies) coupled with quadrupole time-of-flight (AB Sciex TripleTOF 6600). An ACQUITY UPLC BEH Amide column (2.1 mm × 100 mm, 1.7 μm, waters) was utilized for chromatographic separation.

A total of six quality control samples were tested during the experiment. Pooled quality control (QC) samples (generated by taking an equal aliquot of all the samples included in the experiment) were run at the beginning of the sample queue for column conditioning and every ten injections thereafter to assess inconsistencies that are particularly evident in large batch acquisitions in terms of retention time drifts and variation in ion intensity over time.

### Metabolomics data preprocessing and analysis

The raw MS data (wiff.scan files) were converted to MzXML files using ProteoWizard MSConvert before importing into freely available XCMS software. More details are described in the supplemental materials. Seven-fold cross-validation and response permutation testing were used to evaluate the robustness of the model. The variable importance in the projection (VIP) value of each variable in the OPLS-DA model was calculated to indicate its contribution to the classification. Moreover, VIP > 1 and *p* < 0.05 were used to screen significantly changed metabolites. Pearson’s correlation analysis was performed to determine the correlation between the two variables. Fold changes were computed as the ratio of peak area between the two groups.

### Statistical analysis

The processed data were analyzed by the R 4.2.0 program. The beta diversity was visualized by two-dimensional principal coordinates analysis (PCoA) of weighted and unweighted UniFrac distance matrices, and statistical comparisons of groups were performed with the non-parametric MANOVA methods using the Adonis function in vegan R package. Orthogonal partial least-squares discriminant analysis (OPLS−DA) were performed using R program ade4 package and mixOmics package, respectively. To identify distinct taxonomic bacterial biomarkers, the linear discriminant analysis effect size (LefSE) was used, and the linear discriminant analysis (LDA) log score cutoff value was determined to be 2 (Segata et al., 2011). Image construction was performed in GraphPad Prism 7 and the R software package. Mann−Whitney tests were performed to test the significance of differences between groups for Intraperitoneal glucose tolerance test (IPGTT), TNF-α, CXCL-15, IL-6, FD4, weight, food intake, etc. Adjusted *p*-values were obtained using Benjamini–Hochberg correction. Comparisons of different timepoints were performed by repeated measures ANOVA followed by Benjamini−Hochberg correction. Adjusted *p* < 0.05 being considered statistically significant.

### Using FMT to construct a GDM mouse model

**FIGURE 1:**
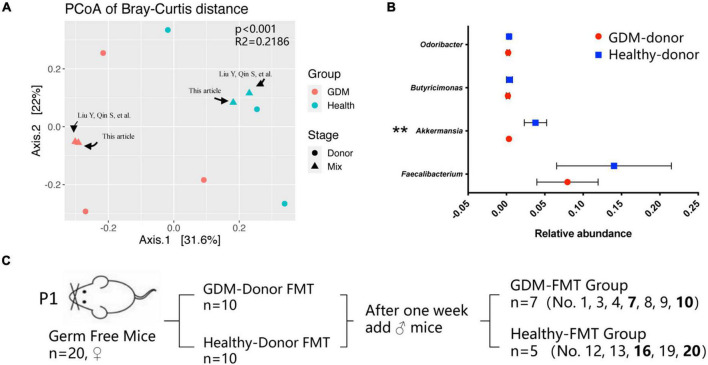
Gestational diabetes mellitus mouse model construction. **(A)** PCoA at the genus level based on Bray−Curtis distance is shown along the first two principal coordinate (PC) axes. Individual samples are represented by blue (healthy pregnancy) and red (GDM), and different stages are represented by triangles (mixed sample) and points (donor sample). **(B)** The bacterial relative abundance of bacteria at the genus level. The data are shown as the median and interquartile range. **(C)** Schematic diagram of the gestational diabetes mellitus mouse model. Number 2 mice in the GDM-FMT group were died naturally for unknown reasons. Numbers 5, 6, 11, 14, 15, 17, and 18 mice were not conceived. **P < 0.01.

We used the two mixed feces used in the past to continue this study, which were derived from three gestational diabetes mellitus patients (GDM donor) and three healthy pregnant women (healthy donor) for germ-free mouse fecal microbiota transplantation. For detailed information, please refer to the article published in 2020 (Liu et al., 2020). Figure 1A shows the clustering difference between the two fecal mix samples and donor feces (*p* < 0.001). The microbial structures of healthy pregnant women and women with GDM were significantly different. The relative abundance of *Akkermansia* in healthy donors was significantly higher than that in GDM donors (Figure 1B, *p* < 0.01), which is basically consistent with the conclusions of our previously published articles and other related studies (Everard et al., 2013; Tilg and Moschen, 2014; Liu et al., 2020).

We selected 20 germ-free female mice to feed on a high-fat diet for 1 week and randomly divided the mice into two groups (10 mice in each group). These two groups of germ-free mice were inoculated by oral gavage of the fecal mixture from the GDM donor and healthy donor, which were used as the experimental group and the control group, respectively. After the FMT mice were reared for 1 week, we recorded the weight of the mice, performed IPGTT, and collected the mouse feces as the basic data of the mice before pregnancy (prepregnancy, Pre). Subsequently, 10 germ-free male mice were mated with female mice for 5 days at a male-to-female ratio of 1:2, and vaginal plug formation in female mice was monitored daily to determine the pregnancy time. The feces of the mice at different pregnancy periods (T1, D4–D6; T2, D11−D13; T3, D18–D20) were collected, and indicators such as feed intake, body weight, IPGTT test, and blood inflammatory factor expression were recorded at the same time. After screening out the data of non-pregnant mice and mice that died naturally, we finally obtained the complete data of seven pregnant mice in the experimental group (GDM-FMT) and five pregnant mice in the control group (healthy-FMT) (Figure 1C).

## Results

### The two groups of mice showed different characteristics of gut microbiota

We performed 16S rRNA gene sequencing analysis on the feces of mice collected at different gestational stages. A total of 3,039,319 reads were obtained by sequencing, and the average number of reads per sample was 63,319. The sample-wise distribution of the reads and the average number of reads per group (stage) are as follows, GDM-FMT-Pre: total 472,014, average 67,431; GDM-FMT-T1: total 416,254, average 59,465; GDM-FMT-T2: total 404,310, average 57,759; GDM-FMT-T3: total 425,410, average 60,773; healthy-FMT-Pre: total 309,764, average 61,953; healthy-FMT-T1: total 338,860, average 67,772; healthy-FMT-T2: total 350,629, average 70,126; healthy-FMT-T3: total 322,078, average 644,156 (Supplementary Table 1).

**FIGURE 2:**
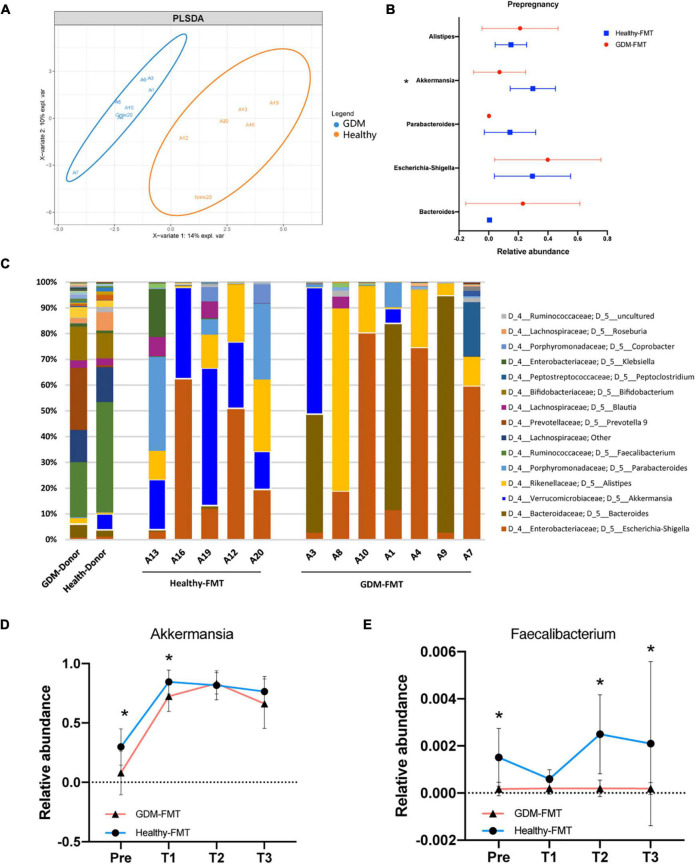
Fecal microbiota transplantation affects the structure of the mouse gut microecology. **(A)** Orthogonal partial least-squares discriminant analysis at the genus level is shown along the first two variate axes. Individual samples are represented by blue (GDM-group) and red (healthy-group), and the two donor data are marked separately in the figure. **(B)** The bacterial relative abundance of bacteria at the genus level. **(C)** The bacterial relative abundance at the genus level after FMT. **(D,E)** The line graph shows the relative abundance of *Akkermansia* and *Faecalibacterium* bacteria between the two groups at different stages. Individual samples are represented by blue lines (healthy-FMT) and red lines (GDM-FMT). The data are shown as the median and interquartile range. *p < 0.05.

We first observed the colonization of intestinal microbiota in two groups of female mice after FMT. The OPLS-DA analysis found that the microbiota characteristics of the two groups of mice tended to be consistent with the donor bacteria (Figure 2A); moreover, the microbiota clusters of the two groups of mice showed significant differences by PCoA analysis (weighted and unweighted) (Supplementary Figure 1). Analysis of the top-five bacteria with relative abundance found that *Akkermansia* in the healthy-FMT group was significantly higher than that of the GDM-FMT group (*p* = 0.05), which was also consistent with the microbiota characteristics of donor bacteria (Figures 2B,C).

Linear discriminant analysis effect size compared the microbial relative abundance of the two groups in different gestational periods and found that *Akkermansia* (Pre), *Eubacterium eligens* group (Pre and T2), *Fusicatenibacter* (Pre), *Christensenellaceae* R_7 group(T1), and *Faecalibacterium* (Pre, T2, and T3) had higher relative abundances in the healthy-FMT group than that in GDM-group (Supplementary Figures S2A and S2D). These bacteria have been shown to be closely related to gestational diabetes (Liu et al., 2020) or anti-inflammatory (Valdez-Palomares et al., 2021). The line graph shows the relative abundance of *Akkermansia*, *Faecalibacterium*, *E. eligens* group, and *Fusicatenibacter* between the two groups. The results showed that the relative abundance of the above bacteria in the healthy-FMT group were higher than that in the control group in nearly four stages (Figures 2D,E and Supplementary Figures 2E,F). In summary, we successfully reshaped gut microecology through FMT in germ-free mice.

### Gestational diabetes mellitus-fecal microbiota transplantation mice had low gut short-chain fatty acids

**FIGURE 3:**
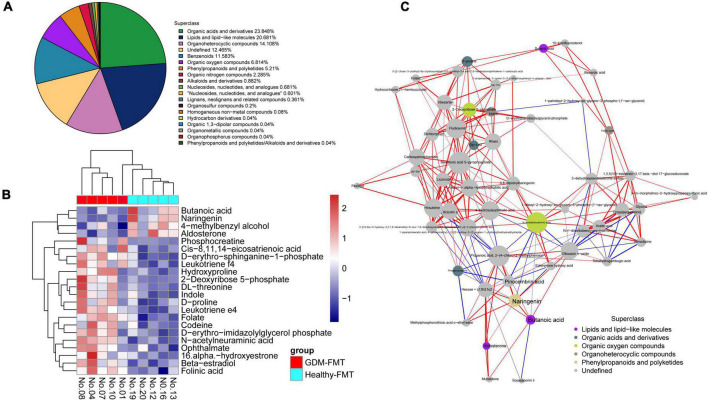
High-resolution non-target metabolomics report. **(A)** The proportion of the number of identified metabolites in each chemical classification. The blocks of different colors in the figure express different chemical classification attribution items, and the percentage represents the percentage of the number of metabolites in the chemical classification attribution entry to the number of all identified metabolites. Metabolites without chemical classification are defined as undefined. **(B)** Significantly different metabolite hierarchical clustering heatmap. Each row represents a differential metabolite, and each column represents a set of samples. Red represents upregulation and blue represents downregulation. Metabolites with similar expression patterns gather under the same cluster on the left. **(C)** The dots in the figure represent significantly different metabolites. The size of the dot is related to the degree of connectivity. The greater the degree, the larger the dot. The color of the line represents correlation, red represents positive correlation, and blue represents negative correlation. The thickness of the line represents the absolute value of the correlation coefficient. The thicker the line, the greater the correlation.

We selected mouse feces (D12) for metabolomics analysis, frightened the mice to excrete fresh feces, and collected 10 (5 in each group) fresh fecal specimens (GDM-FMT: Nos.1, 4, 7, 8, and 10; healthy-FMT: Nos.12, 13, 16, 19, and 20). The combined test results show that organic acids and derivatives (23.848%) and lipids and lipid-like molecules (20.681%) in the metabolites of the intestinal microbiota account for the largest proportion, reflecting the functional characteristics of the gut microbiota (Figure 3A). Metabolite heatmap showing the characteristics of the metabolic clustering of the two groups of bacteria. Butanoic acid (*p* = 0.031, fold changes = 2.25), naringenin (*p* = 0.007, fold changes = 2.21), 4-methylbenzyl alcohol (*p* = 0.050, fold changes = 1.54), and aldosterone (*p* = 0.031, fold changes = 1.39) in the healthy-FMT group were significantly higher than those in the experimental group (GDM-FMT group) (Figure 3B and Supplementary Table 2). Surprisingly, naringenin had a strong positive correlation with pinocembrin (*r* = 0.90), butanoic acid (*r* = 0.88), and propanoic acid (*r* = 0.93) (Figure 3C). The comparison of fecal metabolomics between the two groups shows that both short-chain fatty acids and naringenin play an important role in blood glucose metabolism.

### Impaired glucose tolerance in gestational diabetes mellitus-fecal microbiota transplantation group mice

**FIGURE 4:**
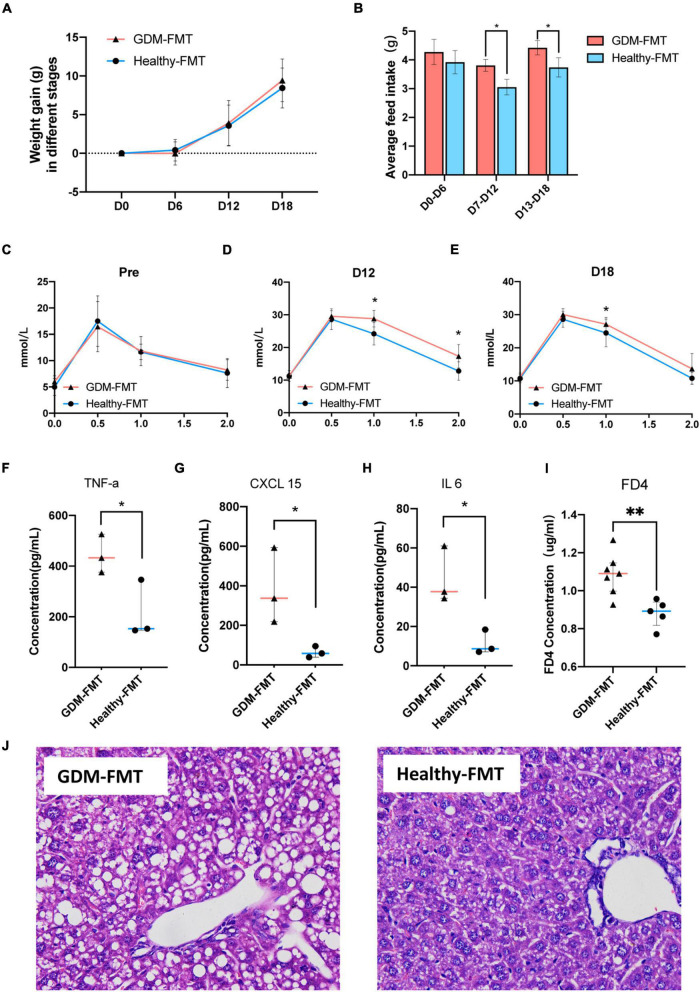
Phenotypic differences between the two groups of mice. **(A)** The line chart shows the body weight of the two groups in different periods. **(B)** The barplot shows the weight gain of the two groups in different time periods. **(C,E)** The line chart shows the results of the IPGTT in different periods. **(F–H)** The scatter plot shows the levels of inflammatory factors (TNF-α, CXCL15, and IL 6) in the plasma of the two groups. The data are shown as the median and interquartile range. **(I)** The scatter plot shows the levels of FD4 in the plasma of the two groups. **(J)** The HE staining diagram of liver cells, microscope 400x field of view. *p < 0.05; **P < 0.01.

Observing the weight gain of the two groups at different gestational ages, no significant difference was found (Figure 4A). However, interestingly, the average feed intake of mice in pregnancy (D0–D6, D7–D12, and D18–D20) was compared, and the food intake of GDM-FMT mice was found to be significantly higher than that of the healthy-FMT group (Figure 4B). To study glucose metabolism during pregnancy, the IPGTT test was performed on mice before mating (D0), 12 days of pregnancy (D12), and 18 days of pregnancy (D18). The 1H and 2H blood glucose levels of the mice in the GDM-FMT group on D12 and D18 were higher than those of the control group (*p* < 0.05) (Figures 4C–E).

The previous studies have shown that GDM is closely related to chronic non-specific inflammation in peripheral blood (Zhu et al., 2013; Mrizak et al., 2014; Xie et al., 2014). To answer whether the GDM-FMT model mice also activated the related inflammatory response, we tested the inflammatory indicators in the plasma of 21-day gestation (D21) mice and found that TNF-α, CXCL 15, and IL6 were expressed in the plasma of GDM-FMT higher than healthy-FMT group (Figure 4F–H, all *p* < 0.05, fold changes = 2.07, 5.96, and 3.90, respectively). In addition, the results of the small intestine permeability test using FD4 suggested that the intestinal permeability of mice in the GDM-FMT group was higher than that of the control group (Figure 4I, *p* = 0.0053, fold changes = 1.23). In addition, we found macrovesicular steatosis was the main type of liver steatosis in GDM-FMT mice, which was more diffuse, involving more parenchymal cells than the control group with scattered little fat droplets. The proportion of hepatic steatosis of GDM-FMT mice was larger than that in the control group(*p* = 0.007), suggesting gut microbiota affects hepatic carbohydrate and lipid metabolism as confirmed by previous studies (Kolodziejczyk et al., 2019; Figure 4J; Supplementary Figures 3, 4). We did not observe more differences between the two groups in the spleen, kidney, and small intestine tissues stained by HE (Supplementary Figure 3). The summary of the current results showed that gut microbial imbalance in the GDM-FMT group guides the activation of inflammatory factors, impairs the barrier function of the small intestine, and leads to impaired glucose tolerance in pregnant mice.

### Abnormal blood glucose metabolism in the offspring of gestational diabetes mellitus-fecal microbiota transplantation group mice

**FIGURE 5:**
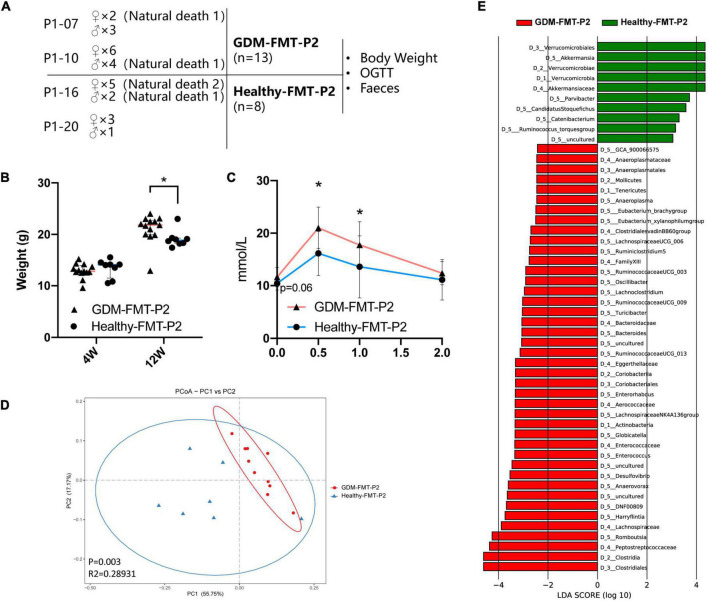
Construction of offspring mouse model and phenotypic characteristics. **(A)** Schematic diagram of the offspring mouse model. **(B)** Scatter plot showing the weight of offspring at different ages. **(C)** The line chart shows the results of the 12-week-old IPGTT. **(D)** PCoA at the genus level based on Bray−Curtis distance is shown along the first two principal coordinate (PC) axes with Adonis *p*-values. **(E)** Through LDA analysis, the LDA scores of the microbial groups with significant effects in different groups are counted, showing the biomarker with significant differences, and the length of the histogram represents the impact of significantly different species. The healthy FMT-P2 group with a positive LDA score is shown in green. The GDM-FMT-P2 group with a negative LDA score is shown in red (cutoff value ≥ 2; *p* < 0.05). The numbers and letters in front of the bacteria represent classifying organisms markers: D_1: represents “Phylum,” D_2: represents “Class,” D_3: represents “Order,” D_4: represents “Family,” D_5: represents “Genus.” *p < 0.05.

The previous studies have shown that the offspring of women with GDM have an increased risk of diabetes (Khan, 2007; Farahvar et al., 2019; Sparks et al., 2022). Colonization of the neonatal microbiota is most affected by the mother’s microbiota (Jakobsson et al., 2014; La Rosa et al., 2014; Cong et al., 2016; Liu et al., 2019). To study the structural characteristics of the gut microecology of the offspring, we randomly selected four pregnant mice (two in each group) to give birth naturally (marked as GDM-FMT-P2 and healthy-FMT-P2). Four weeks postpartum, the pups were separated from the mothers, and female, and male mice were kept separately for eight weeks with a high-fat diet in an SPF environment. The experimental group gave birth to 15 mice (2 died naturally), and the experimental data of 13 mice (7 female mice and 6 male mice) were obtained. The control group gave birth to 11 pups (3 died naturally) and finally obtained experimental data from 8 mice (6 female mice and 2 male mice). The above five mice died naturally within 1 week after delivery (Figure 5A).

We performed 16S rRNA gene sequencing analysis of pup feces. Four mice feces failed to establish the fecal bacteria library, and finally the fecal sequencing information of 17 mice (10 belonged to the GDM-FMT-P2 group and 7 belonged to the healthy-FMT-P2 group) was obtained. A total of 5,058,586 reads were obtained by sequencing, and the average number of reads per sample was 297,564. The sample-wise distribution of the reads and the average number of reads per group are as follows, GDM-FMT-P2: total 2,887,020, average 288702; healthy-FMT-P2: total 2,171,566, average 310,224 (Supplementary Table 1).

Our results suggest that there was no significant difference in the weight of 4-week-old pups between the two groups (*p* > 0.05). However, at 12 weeks of age, the weight of GDM-FMT pups was significantly higher than that of healthy-FMT pups (*p* < 0.05) (Figure 5B). More importantly, the results of the 12-week-old IPGTT showed that the 0.5 and 1-h blood glucose levels in the GMD-FMT-P2 group were higher than those in the healthy-FMT-P2 group (Figure 5C). Comparing the analysis of the gut microbiota of the two groups of offspring, significant differences in bacterial clustering were found (Figure 5D). The LefSe results showed that the relative abundances of *Akkermansia*, *Parvibacter*, *Ruminococcus torques*, *Catenibacterium*, and *Candidatus Stoquefichus* in the offspring of the healthy-FMT-P2 group were higher than those in the offspring of the GDM-FMT-P2 group. In addition, *Anaeroplasma*, *Eubacterium_brachy* group, *Eubacterium_xylanophilum* group, *Lachnospiraceae* UCG 006, *Ruminiclostridium 5*, *Ruminococcaceae* UCG_003, *Oscillibacter*, *Lachnoclostridium*, *Ruminococcaceae* UCG_009, *Turicibacter*, *Bacteroides*, *Ruminococcaceae* UCG_013, *Enterorhabdus*, *Lachnospiraceae* NK4A136 group, *Globicatella*, *Enterococcus*, *Desulfovibrio*, *Anaerovorax*, DNF00809, *Harryflintia*, and *Romboutsia* had higher relative abundances in the GDM-FMT-P2 group (Figure 5E). Therefore, the above results indicate that maternal intestinal microecology directly affects the intestinal microenvironment and the growth and development of offspring.

## Discussion

In this FMT pregnancy mouse model study, the transplantation of fecal microbiota to GF mice showed different gut microbiota colonization patterns, and elevated blood glucose was induced in GF pregnancy mice receiving GDM microbiota. The influence of the gut microbiota continued on the offspring of mice and was also reflected in the increase in blood glucose and food intake in the experimental group. *Akkermansia* and *Faecalibacterium* were significantly lower in GDM-FMT mice than in the control group, which may be related to the content of short-chain fatty acids and naringenin in feces.

### The state of pregnancy overturns the current gut microbial environment

The structure of the gut microbiota of mice changes dramatically before and during pregnancy. Our article published in 2021 introduced the changes in the intestinal microbiota of healthy women during pregnancy (early trimester, second trimester, and third trimester) and 6 weeks postpartum and found great changes in the gut microbiota during pregnancy and postpartum (Qin et al., 2021). In this experiment, we found that the mice had significant changes after pregnancy compared with before pregnancy, indicating that only the state of pregnancy (excluding the influencing factors of dietary changes and lifestyle during pregnancy) may be enough to change the intestinal microbial environment drastically. Moreover, the relative abundance of *Akkermansia* bacteria in all pregnant mice was significantly increased, indicating that a certain mechanism of pregnant individuals can promote the colonization and growth of *Akkermansia* bacteria, and this promotion has been more clearly demonstrated in healthy pregnant individuals. However, our results also showed that the *Faecalibacterium* bacteria did not have the above changes.

In our previous article (Liu et al., 2020), we found that GDM patients have intestinal microecology changed, the relative abundance of *Akkermansia* bacteria decreased, and the relative abundance of *Blautia* and *Faecalibacterium* increased. *Akkermansia* bacteria play an extremely important anti-inflammatory and hypoglycemic role during pregnancy. However, we found that *Faecalibacterium* had three periods of increased relative abundance in the healthy FMT group (Pre, T2, and T3). Our previous research suggests that the relative abundance of *Faecalibacterium* in GDM was higher than that in healthy controls in the second trimester. Reviewing the literature, *Faecalibacterium* is a butyrate producer with anti-inflammatory effects and has been shown previously to be reduced in the third trimester of healthy women compared to earlier pregnancy (Sokol et al., 2008; Koren et al., 2012; Crusell et al., 2018).

### Metabolic characteristics of the gut microbiota

We performed metabolomics analysis on the feces of the two groups of pregnant mice and found that the levels of butyric acid and naringenin in the GDM-FMT group were significantly reduced. Butanoic acid is a kind of SCFAs that mainly includes acetic acid, propionic acid, and butanoic acid. Most SCFAs are the final product of bacterial fermentation. Short-chain fatty acids are important players in the interaction between the host and gut microbiota (Tsvetikova and Koshel, 2020) and have a variety of functions to maintain human health; for example, as a special nutrient supply and energy production component of the intestinal epithelium (Chen et al., 2017), SCFAs protect the intestinal mucosal barrier, reduce the level of inflammation in the body (Yang et al., 2020), and enhance gastrointestinal motility and function (Manichanh et al., 2012).

Naringenin is a citrus flavonoid that possesses a variety of biological activities. Studies have shown that naringenin has anti-bacterial (Rauha et al., 2000; Zeng et al., 2018), anti-inflammatory (Bodet et al., 2008; Yang et al., 2021), and scavenging free radicals and anti-oxidant effects (Yu et al., 2005; Rashmi et al., 2018). In non-gestational tissues, naringenin and apigenin inhibit proinflammatory mediators such as PGE2, cyclooxygenase-2 (COX-2) and TNF-α and suppress NF-Kb (Nicholas et al., 2007; Park et al., 2012). Researchers at the University of Melbourne treated the placenta, fetal membranes and myometrium with curcumin, naringenin and apigenin in the presence of lipopolysaccharide (LPS) or interleukin (IL)-1β. In the placental and fetal membranes, all treatments significantly reduced LPS-stimulated release and gene expression of the proinflammatory cytokines IL-6 and IL-8. In myometrial cells, all treatments attenuated IL-1β-induced COX-2 expression, release of PGE2 and PGF2α and expression and activity of MMP-9. Naringenin significantly attenuated IL-1β-induced IL-6 and IL-8 mRNA expression and release, and there was no effect on curcumin and apigenin (Lim et al., 2013). In addition, we found that naringenin has a strong positive correlation with pinocembrin [also proven to have anti-inflammatory effects (Sala et al., 2003; Wang et al., 2013; Poblocka-Olech et al., 2019)], butanoic acid and propanoic acid.

The content of naringenin in the intestinal of the two groups of mice consuming the same diet was significantly different, which may be the consequence of the effect of the gut microecology. Naringinase is an enzyme that has a wide occurrence in nature. Many natural glycosides(citrus flavonoids), including naringin, rutin, quercitrin, hesperidin, diosgene, and terphenyl glycosides, contain terminal α-rhamnose and β-glucose can act as substrates of naringinase. In humans, naringinase is found in the liver and rapidly metabolizes naringin into naringenin (Ribeiro, 2011). Therefore, we believe that the difference in intestinal naringenin content may be related to gut microecology and the biological activity of naringinase, which requires more in-depth research. Understanding the relationship between them may be important for clinical intervention in the intestinal microecology of pregnant women with gestational diabetes.

### Effect of offspring mouse gut microbiota colonization, growth, and developmental changes

In this study, we studied the characteristics of bacterial colonization in offspring mice. We reared the offspring in a sterile environment until 4-weeks old and then divided them into SPF environments. After the offspring had constructed their own gut microbiota, we compared the two groups of gut bacteria and found that in healthy-FMT offspring, the relative abundance of *Akkermansia* bacteria, *Parvibacter*, *and Ruminococcus torques* group was significantly higher than that in the GDM-FMT group. These bacteria have been shown to be closely associated with blood sugar, anti-inflammatory and lipid metabolism (Song et al., 2019; Wu et al., 2022), and produce extracellular glycosidases (Hoskins et al., 1985). In contrast, *Romboutsia*, *Lachnospiraceae* UCG 006, *Oscillibacter*, *Lachnoclostridium*, and *Harryflintia* were more abundant in the intestines of GDM-FMT offspring mice. The differences in the microbiota of the offspring mice are reflected in their growth and development. Our research found that the weight gain and blood sugar levels of GDM offspring mice were higher than those of the control group. This shows that the colonization and future growth and development of the offspring’s intestinal microbiota are closely related to the intestinal microbiota of the mother.

The above analysis results are based on the relevant data of 21 offspring mice. These pups were derived from only four parent mice (two in each group), making the observed differences between offspring likely due to mothers between random variability or cage effects. During the experiment, we took various measures to make the data more reflective of the actual situation: (1) Performed the experiments in a sterile environment; (2) After successful pregnancy, all pregnant mice were kept in single cages; (3) Randomly selected mother mice for delivery.

## Conclusion

**FIGURE 6:**
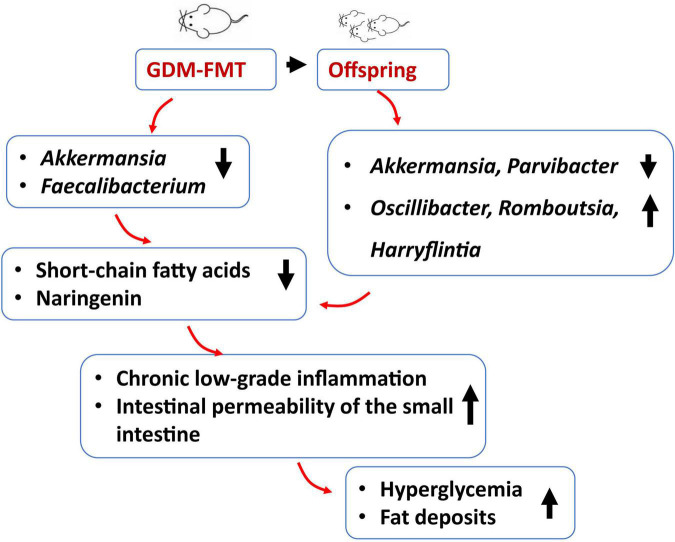
Schematic diagram of GDM gut microecological characteristics.

Here, we designed this experiment to investigate in-depth the effect of gut microbiota in humans with GDM on blood glucose in pregnant mice and their impact on colonization of the microbiota of offspring. Mice are fertilized after FMT to simulate human pregnancy. Various abnormalities occurred in pregnant mice in the GDM-FMT group compared with the control group: a lower bacterial relative abundance of *Akkermansia* and *Faecalibacterium* in the gut, elevated blood glucose, high expression of inflammatory factors, and liver fat deposition. The offspring mice of this group were also had higher blood glucose levels (Figure 6). Based on the above results, we believe that the disturbance of gut microbiota in mother mice affects the development of the offspring, which provides a stronger basis and higher expectations for the intervention necessity and methods of the gut microbiota in pregnant women.

There are some shortcomings in this study. (1) Feed intake is affected by the number of fetuses in pregnant mice. It is generally believed that the nutritional intake and weight gain of multiple pregnancies are higher than that of singleton pregnancies. Feed intake is affected by the number of female mouse embryos, but we are missing this part of the data. (2) Despite our efforts to reduce between-group differences, the observed differences between offspring may still be affected by random variability between mothers or cage effects, so further experiments are warranted to validate the above results.

## Data availability statement

Data that support the findings of this study are available under BioProject ID PRJNA836862. https://www.ncbi.nlm.nih.gov/bioproject/PRJNA836862.

## Ethics statement

The animal study was reviewed and approved by Peking University Hospital Laboratory Animal Welfare Ethics Review Committee. Written informed consent was obtained from the owners for the participation of their animals in this study. Written informed consent was obtained from the individual(s) for the publication of any potentially identifiable images or data included in this article.

## Author contributions

SQ designed and performed the experiments and wrote the original draft. HY conceptualized and supervised the experiments. YW, SW, and BN conducted data analysis. HY conducted a writing review. All authors contributed to the article, approved the submitted version, and have read and agreed to the published version of the manuscript.
