# Supplementary material for: Gut microbiota in women with gestational diabetes mellitus has potential impact on metabolism in pregnant mice and their offspring
Source: Front Microbiol. 2022 Aug 5;13:870422. doi: 10.3389/fmicb.2022.870422 (PMC9389115; doi:10.3389/fmicb.2022.870422)
Supplement: Supplementary file 1 [file Data_Sheet_1.docx]

**Supplementary Materials:**

Methods S1. UHPLC-Q-TOF/MS parameters for metabolic profiling acquisition

In both ESI positive and negative modes, the mobile phase contained A=25 mM ammonium acetate and 25 mM ammonium hydroxide in water and B= acetonitrile. The gradient was 85% B for 1 min and was linearly reduced to 65% in 11 min, reduced to 40% in 0.1 min and kept for 4 min, and then increased to 85% in 0.1 min, with a 5 min re-equilibration period employed. For Q-TOF analysis, the ESI source conditions were set as follows: Ion Source Gas1 (Gas1) as 60, Ion Source Gas2 (Gas2) as 60, curtain gas (CUR) as 30, source temperature: 600℃, and IonSpray Voltage Floating (ISVF) ±5500 V. In MS-only acquisition, the instrument was set to acquire over the m/z range of 60. In auto MS/MS acquisition, the instrument was set to acquire over the m/z range of 25-1000 Da, and the accumulation time for the product ion scan was set at 0.05 s/spectra. The product ion scan was acquired using information-dependent acquisition (IDA) with high sensitivity mode selected. The parameters were set as follows: the collision energy (CE) was fixed at 35 V with ±15 eV, declustering potential (DP), 60 V (+) and −60 V (−); excluding isotopes within 4 Da, candidate ions to monitor per cycle: 10.

Methods S2. The parameters for data processing

The raw MS data (wiff.scan files) were converted to MzXML files using ProteoWizard MSConvert before importing into freely available XCMS software. For peak picking, the following parameters were used: centWave m/z = 10 ppm, peak width = c (10, 60), and prefilter = c (10, 100). For peak grouping, bw = 5, mzwid = 0.025, and minfrac = 0.5 were used. CAMERA (Collection of Algorithms of MEtabolite pRofile Annotation) was used for annotation of isotopes and adducts. In the extracted ion features, only the variables having more than 50% of the nonzero measurement values in at least one group were kept. Compound identification of metabolites was performed by comparing the accuracy m/z value (<10 ppm) and MS/MS spectra with an in-house database established with available authentic standards.

Methods S3. FD4 test

Intestinal permeability assay in mice using the sigma FD4 (FITC–Dextran) kit. First, about 200ul (8mg) of FD4 (0.4mg/g weight) was drawn using a 1mL syringe and introduced into the stomach of each mouse by gavage. Tail blood collection (about 50-100ul) was then performed 1H after gavage. The blood was stored in a red coagulation-promoting tube, and after the blood was coagulated, the serum was centrifuged for detection. After the blood was diluted, the serum concentration of FITC-dextran was measured using an excitation wavelength of 490 nm and an emission wavelength of 530 nm.

Methods S4. HE staining

1. Place the mouse tissue in the labeled numbered cassette.

2. Dehydration, transparency, wax dipping

1) 70% alcohol: 1.5h

2) 80% alcohol: 30min

3) 90% alcohol: 30min

4) 95% alcoholⅠ: 30min

5) 95% alcoholⅡ: 30min

6) 100% alcoholⅠ: 30min

7) 100% alcoholⅡ: 30min

8) Xylene anhydrous ethanol mixture (1:1)：10min

9) Xylene Ⅰ: 5min

10) Xylene Ⅱ: 5min

11) 58-60℃ paraffin Ⅰ

12) 58-60℃ paraffin Ⅱ

3. Embedding

1) Open the cover of the embedding box. Use tweezers to clip the tissue out and place it on the wax table. The bottom of the embedding box is also placed on one side of the wax table, and then use tweezers to remove the metal mold in the wax tank. Take it out and place it on the wax table.

2) Add a layer of paraffin liquid to the metal mold. When the surface of the liquid turns white, use tweezers to pick up the tissue and put it into the paraffin liquid. After waiting for 3s, place the white embedding box on the metal mold, and then put it into the mold. Add fresh paraffin to completely cover the tissue, and stop adding paraffin when the liquid surface is parallel to the edge of the cassette.

3) Translate the metal mold that has dripped paraffin into the cooling table on the right hand, and place the wax block on the cooling table for 30 minutes.

4) Put the wax block with the metal mold in the -20℃ refrigerator for 10 minutes, and separate the metal mold and the wax block.

5) Put the embedded paraffin block in a ziplock bag and place it at 4℃ overnight.

4. Slicing

1) Take the wax block out of the 4°C refrigerator, and put it in the -20°C refrigerator to pre-cool for 30 minutes.

2) During the pre-cooling process, prepare the slide and mark the bottom area of the slide with a pencil; also fix the blade on the microtome to make sure that the microtome is "locked"; Turn on the tablet machine, add 3/4 of ultrapure water to the basin, and wait until the water temperature reaches 41°C.

3) The pre-cooled wax block is fixed on the microtome, and the trimming thickness is adjusted to 10 μm. When it is found that the entire shape of the tissue can be cut on the same slice, the trimming thickness is adjusted to 4 μm.

4) Take a corner of the cut slice with tweezers and move it to the water basin of the slicer. After the slice is flattened, take out the slice with a marked glass slide and observe under a microscope whether the slice is fully flattened. After confirmation, put the slices on the baking machine, and set the temperature of the baking machine to 60°C.

5. Bake the slices in a 60°C oven for 4 hours.

6. HE staining

1) Dewaxing. Xylene I 15min, Xylene II 15min, 100% ethanol 5min, 95% ethanol 5min, 80% ethanol 5min, 70% ethanol 5min, ultrapure water I 5min, ultrapure water II 5min.

2) Stain with hematoxylin solution for 1 min.

3) Rinse 3 times with ultrapure water.

4) The tap water returns to blue for 10 minutes.

5) 0.5% eosin staining solution for 10s.

6) Rinse 5 times with tap water.

7) Dry the slices in a 60°C oven.

8) Seal the slide with neutral resin.

**Supplementary Figures:**

**Figure S1** PCoA at the genus level in different stage and group based on Bray−Curtis distance is shown along the first two principal coordinate (PC) axes with P values.

**Figure S2** (a-d) Through LDA analysis, the LDA scores of the microbial groups with significant effects in different groups were counted, showing the biomarker with significant differences, and the length of the histogram represents the impact of significantly different species. The healthy FMT group with a positive LDA score is shown in red. The GDM-FMT group with a negative LDA score is shown in green (cutoff value≥2, p<0.05). (e-f) The line graph shows the relative abundance of *Eubacterium Eligens* group and *Fusicatenibacter* bacteria between the two groups at different stages. Individual samples are represented by blue lines (Healthy-FMT) and red lines (GDM-FMT). The data are shown as the median and InterQuartile Range (IQR).

**
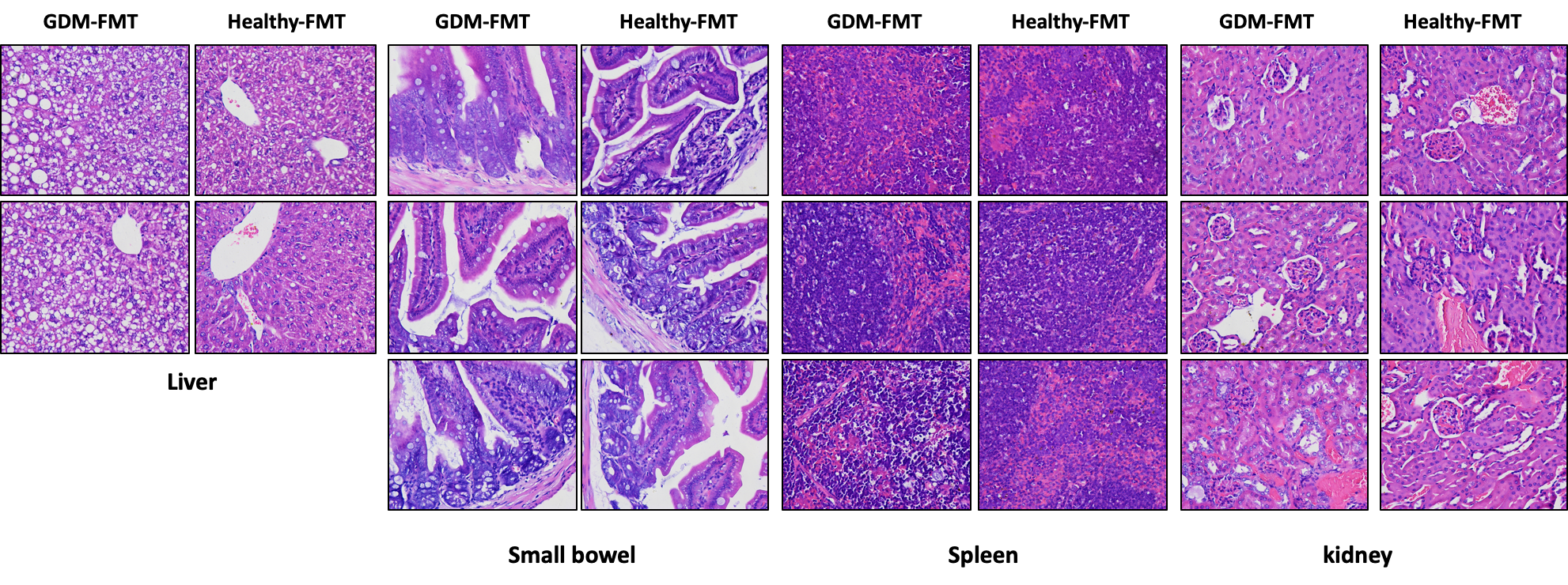
**

**Figure S3** HE staining diagram (liver cells, small bowel, spleen and kidney), microscope 400x field of view.

**Figure S4** The scatter plot shows the percentage of fat vesicles in the liver cells of the two groups of mice.
